# Damage Identification of Truss Bridge Under Temperature Variations Based on Stiffness Separation Method

**DOI:** 10.3390/s26123791

**Published:** 2026-06-14

**Authors:** Feng Xiao, Yijing Gong, Yujiang Xiang

**Affiliations:** 1 School of Safety Science and Engineering, Nanjing University of Science and Technology, Nanjing 210094, China; 2 School of Mechanical and Aerospace Engineering, Oklahoma State University, Stillwater, OK 74078, USA

**Keywords:** damage identification, temperature variation, stiffness separation method, truss bridge, joint damage–temperature identification

## Abstract

Structures are continuously subjected to the combined effects of temperature variations and other environmental factors during service, leading to changes in their structural responses and severely compromising the accuracy of damage identification. To address this temperature-induced interference, this study proposes a damage identification method that jointly identifies damage and temperature parameters. Temperature variations are treated as unknown parameters and identified simultaneously with the damage parameters. The inverse problem is defined by an objective function that quantifies the discrepancy between measured and analytical strains. To enable the analysis of large-scale structures, a stiffness separation method accounting for temperature variation is introduced, which divides the structure into substructures for damage identification. This method is numerically demonstrated through a case study of a steel truss bridge, and the applicability of the combined temperature identification and stiffness separation method is evaluated under different temperature conditions.

## 1. Introduction

In steel truss bridges, thermal strains from ambient temperature variations can conceal strain changes caused by structural damage in measurements. Even in the absence of operational loads, thermal expansion and contraction of members generate significant structural responses that can mask damage signals and lead to false or missed detections [1,2,3]. The structure is subjected to complex temperature variations due to solar radiation, shading, etc. These differences are magnified by daily or seasonal changes [4]. As a result, temperature-induced strain signals are superimposed on the strain changes due to damage and load, interfering with the accuracy of identification models.

Researchers recognize that accounting for temperature is crucial for reliable damage identification. Zhu et al. [5] used long-term monitoring data to show that temperature variations significantly affect steel truss bridge performance. Then, Zhu et al. [6] further demonstrated that temperature-induced responses are highly sensitive to bridge properties, especially bearing performance in long-span arch bridges. Zhang et al. [7] noted that complex cross-sections and increased spans can generate non-uniform temperature fields and localized thermal effects, leading to excessive thermal stresses and potential damage. Zhou et al. [8] studied the static temperature response of long-span steel box girder suspension bridges using simulations and field measurements, revealing that main cables and the bridge deck exert the most significant temperature influence.

Some studies treat temperature as an influencing factor and conduct damage identification [9,10,11]. Dinh et al. [9] employed the Chaos Game Optimization (CGO) algorithm under incomplete, noisy, and temperature variation conditions. An objective function combining modal assurance criteria and flexibility residuals transformed damage identification into an optimization problem. Numerical examples demonstrate effective location and quantification of damage in steel and aluminum structures. Kourehli et al. [10] proposed a damage detection method under varying temperature conditions by integrating modal data with the Marine Predator Algorithm. Its effectiveness was validated under scenarios involving independent damage, temperature-coupled damage, and modal data containing noise. Gu et al. [11] proposed a two-step method combining a multilayer neural network with novelty detection, which uses natural frequencies and temperature measurements to distinguish damage-induced changes from temperature effects.

The present study focuses on the joint damage–temperature identification and, more specifically, on methods that treat both temperature and damage as unknowns. For instance, Meruane and Heylen [12] distinguished temperature effects from actual damage using modal parameters and employed a parallel genetic algorithm to solve the inverse problem for updating both temperature and damage parameters. Huang et al. [13,14,15] established temperature-dependent elastic modulus relationships and constructed objective functions integrating natural frequencies, mode shapes, and modal strain energy. The studies addressed the joint identification of damage and temperature variations using optimization-based approaches.

Overall, for large-scale structures, damage identification under temperature variations requires further study. Damage identification approaches can be categorized into physics-based [16,17,18,19] and data-driven methods [20,21,22,23]. This study is physics-based and adopts the static-based approach for damage identification. Static responses are sensitive to local stiffness changes and have a direct physical relationship with the structural stiffness matrix. The challenges in identifying damage in large-scale structures primarily manifest in the following aspects: the high dimensionality of the system, the multitude of unknown parameters, and the masking effect of environmental variations, especially temperature. The stiffness separation method [24,25,26,27] addresses this by structuring the model into substructures and analyzing each separately. This decomposition reduces computational complexity and decreases the number of simultaneous unknown parameters.

In summary, to address the challenge of applying joint damage–temperature identification to large-scale structures, this study introduces a method that couples the joint damage–temperature identification approach with the stiffness separation method. The proposed method directly extracts the substructural stiffness submatrix from the global system, enabling independent parameter identification for each substructure under temperature variation. By decomposing the global problem into substructures, the proposed method reduces computational complexity and offers a solution for scenarios where temperature sensors are scarce, or temperature measurement errors are significant.

## 2. Damage Identification Under Temperature Variations

This section presents the method for joint identification of damage and temperature variations. To address the challenge of decoupling thermal effects from structural damage, the damage identification under temperature variations is studied. This method treats temperature variations as unknown parameters and identifies them jointly with the damage parameters through an optimization process. The foundation of this approach lies in establishing the structural stiffness equation that incorporates both temperature impacts and damage-induced stiffness reduction. The derivation begins with the elemental stiffness matrix.

The member stiffness matrix k in global coordinates is given by:

(1) $$k\;=\;T^{T}k^{\prime }T$$

where T and $T^{T}$ are the displacement transformation matrix and its transpose, respectively, and $k^{\prime }$ is the member stiffness matrix in local coordinates. The global force–displacement relationship under temperature changes is expressed as:

(2) $$Q\;=\;\mathrm{KD}\;+{\;Q}_{0}\;$$

where Q is global forces and D is global displacements of the structure. $Q_{0}$ denotes the initial fixed-end forces for the entire truss, caused by temperature variations in the members. Partitioning the global forces and displacements in Equation (2) into known and unknown quantities leads to Equation (3):

(3) $$\left[\begin{array}{c} Q_{k}\\ Q_{u} \end{array}\right]=\left[\begin{array}{cc} K_{11} & K_{12}\\ K_{21} & K_{22} \end{array}\right]\left[\begin{array}{c} D_{u}\\ D_{k} \end{array}\right]+\left[\begin{array}{c} {\left(Q_{k}\right)}_{0}\\ {\left(Q_{u}\right)}_{0} \end{array}\right]$$

In this study, damage is simulated by reducing the cross-sectional area of the members, which represents a reduction in their stiffness. The damaged areas are represented by the vector $A_{d}$, and the temperature variations are represented by the vector ΔT. Both are treated as unknown parameters to be identified. Thus, the structural response depends on both the damaged areas $A_{d}$ and the temperature variations ΔT. Consequently, the nodal displacements $D_{u}$ are functions of $A_{d}$ and ΔT, as detailed in Equation (4).

(4) $$D_{u}\left(A_{d},\;\Delta T\right)={\left[K_{11}\left(A_{d\;}\right)\right]}^{-1}\left[Q_{k}-K_{12}\left(A_{d}\;\right)D_{k}-{\left(Q_{k}\right)}_{0}\left(A_{d}\;,\;\Delta T\right)\right]\;\;\;$$

The analytical strain is derived from the nodal displacements of its two ends. Thus, the objective function is constructed from the discrepancy between measured strain $\varepsilon_{m}^{i}$ and analytical strain $\varepsilon_{a}^{i}(A_{d},\Delta T)$:

(5) $$f\left(A_{d},\;\Delta T\right)=\sum_{i=1}^{M}{\left[\varepsilon_{m}^{i}-\varepsilon_{a}^{i}\left(A_{d},\;\Delta T\right)\right]}^{2}$$

In Equation (5), i denotes the i-th measurement point and M is the total number of the measurement points. Optimal parameters are identified through minimization of the objective function. In this study, the pattern search algorithm [28,29,30] is employed to solve this optimization problem. When the objective function approaches zero, the identified $A_{d}$ and ΔT yield analytical strains that match the measured strains and can therefore be regarded as estimates of the damaged and thermal state.

To assess the precision of the damage identification, the mean relative error (MRE) is employed.

(6) $$MRE=\frac{1}{N}\sum_{j=1}^{N}\left|\frac{A_{d_{j}}^{\prime }-A_{d_{j}}^{*}}{A_{d_{j}}^{\prime }}\right|$$

where N represents the number of the damaged members. $A_{d_{j}}^{\prime }$ and $A_{d_{j}}^{*}$ represent the actual damaged and identified cross-sectional area, respectively.

## 3. Truss Structure Damage Identification

To validate the effectiveness of the joint damage–temperature identification method presented in Section 2, a case study, referred to as Case A, is conducted using a Howe-style truss. Different damage scenarios and temperature variation conditions are designed to evaluate the accuracy and robustness of the proposed method in simultaneously identifying damage and temperature parameters.

**Figure 1:**
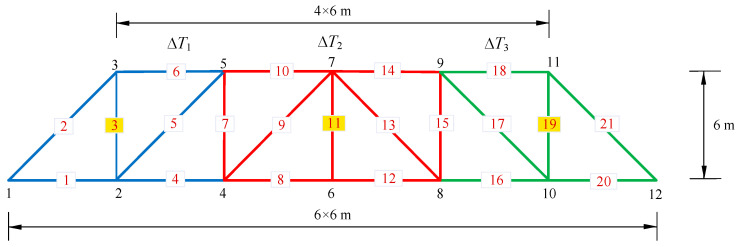
Howe-style truss structure with member and node numbering.

The truss model, shown in Figure 1, comprises 12 nodes and 21 members, with a total length of 36 m and a height of 6 m. The numbers adjacent to the joints indicate node labels, and the numbers placed along members indicate member labels. The members are grouped into three temperature zones, distinguished by three colors. The structure has pin supports at node 1 and roller supports at node 12. The temperature zones are defined as follows: members 1–6 belong to Zone 1 with a temperature change in $\Delta T_{1}$; members 7–15 form Zone 2 with a temperature change in $\Delta T_{2}$; and members 16–21 constitute Zone 3 with a temperature change in $\Delta T_{3}$.

The material elastic modulus is 210 GPa, and the thermal expansion coefficient α = 1.17 × 10^−5^/°C. Each member has the same cross-sectional area, and the initial cross-sectional area is 0.02 m^2^. A static load of 100 kN is applied vertically downward at node 6. In this study, a reference temperature of 20 °C is set as the baseline for all temperature variation scenarios.

Three damage scenarios, Cases A-1 to A-3, are investigated, with the corresponding reductions in cross-sectional area and the prescribed temperature variations listed in Table 1 and Table 2, respectively. With damage present at multiple locations and across three distinct temperature zones, a total of six parameters, three damage-related and three temperature parameters, require identification. The strain measurements used in this study are derived from simulated damage scenarios and taken on the damaged members in each case. Additionally, in this study, to account for the introduction of temperature as an unknown parameter, extra strain gauges are placed within each temperature variation zone. Specifically, strain gauges are placed on members 3, 11, and 19, which correspond to the three distinct temperature zones. The locations of these additional sensors are highlighted in yellow in the structural diagram.

In this study, for the optimization process, the initial values of the cross-sectional areas are set to their undamaged values, and the initial temperature variations are set to 0 °C. Additionally, the cross-sectional area of damaged members is constrained between 0 and its initial value. The temperature variation is given a wide range of −60 °C to +60 °C to avoid relying on restrictive prior bounds.

**Figure 2:**
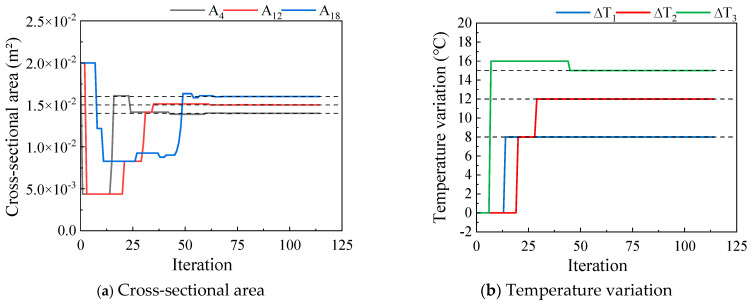
Damage identification results for Case A-1.

**Figure 3:**
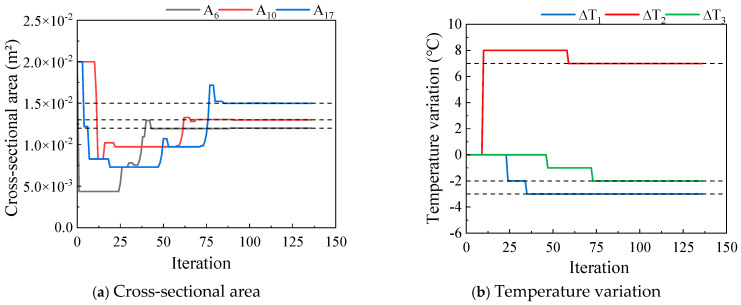
Damage identification results for Case A-2.

**Figure 4:**
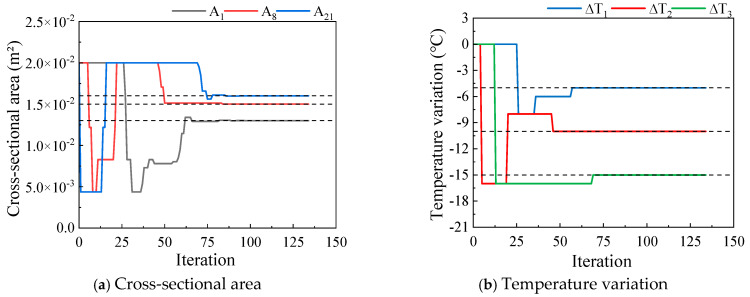
Damage identification results for Case A-3.

Figure 2, Figure 3 and Figure 4 illustrate the identification results for Case A-1, A-2, and A-3, respectively. The dashed lines represent the true values of the parameters. When temperature is incorporated as an unknown parameter, Figure 2a, Figure 3a and Figure 4a show the cross-sectional area parameters converge to the true values. Simultaneously, Figure 2b, Figure 3b and Figure 4b show that the three temperature variation parameters also converge to their preset values. Both the damage and thermal states are accurately identified.

**Figure 5:**
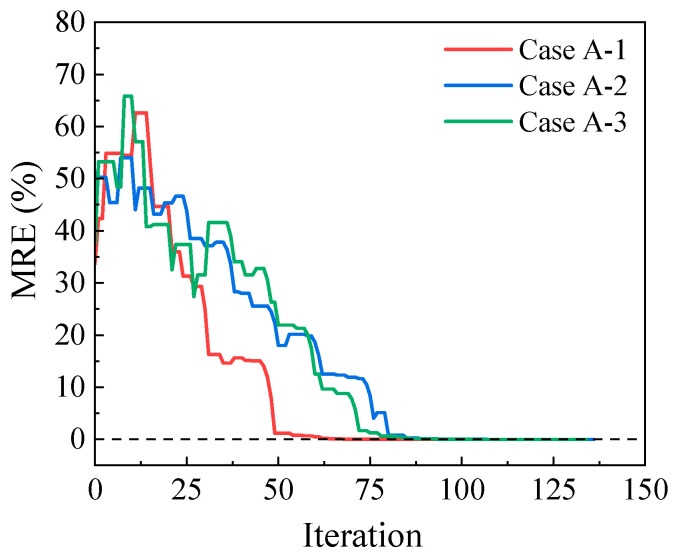
MRE results for Case A.

Figure 5 shows the MRE results for the three cases, as defined in Equation (6). All cases’ MRE values approach zero, indicating that the identified cross-sectional areas closely match the actual damaged values. This demonstrates that the joint identification method can accurately identify damage parameters while simultaneously estimating unknown temperature variations, effectively decoupling the thermal effects from structural damage. The results observed across different damage and temperature scenarios confirm that the proposed method enables accurate damage identification under varying temperature conditions without requiring independent temperature measurements.

## 4. Stiffness Separation Method

To extend the parameter identification method presented in Section 2 to large-scale structures, the stiffness separation method [24,25,26,27] is employed to reduce computational complexity. This section details the mathematical formulation of this method. The core idea involves decomposing the global structure into manageable substructures. The force–displacement relation for each substructure is derived from the global system by extracting the degrees of freedom (DOFs) that are relevant to the substructure.

First, the structural nodes together with their associated DOFs are numbered. The global stiffness matrix is formulated as in Section 2. Assuming the structure has n DOFs, these quantities are expressed as:

(7) $$\begin{array}{c} D\;=\;{\left[D_{1},\;D_{2},\;{\cdots ,\;D}_{n}\right]}^{T},\\ Q\;=\;{\left[Q_{1},\;Q_{2},\;{\cdots ,\;Q}_{n}\right]}^{T},\\ Q_{0}\;=\;{\left[Q_{01},\;Q_{02},\;{\cdots ,\;Q}_{0n}\right]}^{T},\\ K\;=\left[\begin{array}{ccc} \begin{array}{cc} k_{11} & k_{12}\\ k_{21} & k_{22} \end{array} & \ldots & \begin{array}{c} k_{1n}\\ k_{2n} \end{array}\\ \vdots & \ddots & \vdots \\ \begin{array}{cc} k_{n1} & k_{n2} \end{array} & \cdots & k_{\mathrm{nn}} \end{array}\right]. \end{array}$$

For a substructure, let p be the number of unknown displacements and m be the number of non-zero displacements. Define the vectors B and U as the DOFs of unknown and non-zero displacements, respectively. The substructure matrices and vectors, including $K_{p\times m}$, $D_{m}$, $Q_{p}$, and $Q_{0p}$ are extracted from the global matrices and vectors of the structural system according to B and U:

(8) $$\begin{array}{c} B=[b_{1},b_{2},\ldots ,b_{p}],\\ U=\left[u_{1},u_{2},\ldots ,u_{m}\right],\\ D_{m}={\left[D_{u_{1}},D_{u_{2}}{,\ldots ,\;D}_{u_{m}}\right]}^{T},\\ Q_{p}={\left[Q_{b_{1}},Q_{b_{2}}{,\ldots ,\;Q}_{b_{p}}\right]}^{T},\\ Q_{0p}={\left[Q_{0b_{1}},Q_{0b_{2}}{,\ldots ,\;Q}_{0b_{p}}\right]}^{T},\\ K_{p\times m}=\left[\begin{array}{ccc} \begin{array}{cc} k_{b_{1},u_{1}} & k_{b_{1},u_{2}}\\ k_{b_{2},u_{1}} & k_{b_{2},u_{2}} \end{array} & \ldots & \begin{array}{c} k_{b_{1},u_{m}}\\ k_{b_{2},u_{m}} \end{array}\\ \vdots & \ddots & \vdots \\ \begin{array}{cc} k_{b_{p},u_{1}} & k_{b_{p},u_{2}} \end{array} & \ldots & k_{b_{p},u_{m}} \end{array}\right]. \end{array}$$

The substructure force–displacement relation is then given by:

(9) $$Q_{p}={\;K}_{p\times m}D_{m}\;+\;Q_{0p}$$

$K_{p\times m}$ denotes the sub-stiffness matrix containing unknown damage parameters, and $Q_{0p}$ includes unknown temperature variations. Solving Equation (9) yields analytical displacements containing the unknown parameters, enabling the construction of an objective function for parameter identification through optimization. Compared with global identification, this substructuring approach can reduce the number of unknown parameters in the objective function. Moreover, each substructural stiffness matrix has a lower dimension than the global stiffness matrix.

## 5. Damage Identification Based on the Stiffness Separation Method

To demonstrate the applicability of the stiffness separation method in conjunction with joint damage–temperature identification, this section presents a case study on a steel truss bridge—the Benniu Bridge. The method is implemented using two distinct substructuring strategies: vertical separation and V-shaped separation, which are referred to as Case B and Case C, respectively. Each case is further evaluated under different temperature variation scenarios to assess the accuracy of the proposed approach in handling large-scale structures under temperature variations.

**Figure 6:**
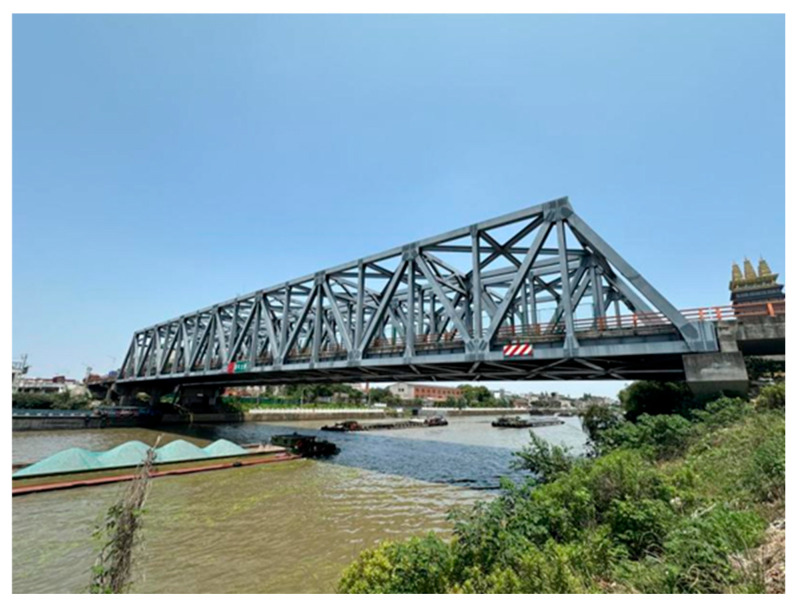
Photograph of Benniu Bridge.

**Figure 7:**
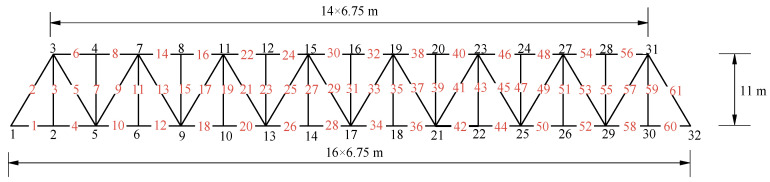
Schematic of simplified structure.

The Benniu Bridge is a 108-m single-span steel truss bridge with a Warren-type main truss, shown in Figure 6. A simplified model of the main truss is developed for analysis (Figure 7). The model comprises 32 nodes and 61 elements, with pin and roller supports at nodes 1 and 32, respectively. Material properties are set as: the elasticity modulus is 210 GPa and thermal expansion coefficient α = 1.17 × 10^−5^/°C. To excite the structure, a set of seven concentrated forces, each with a magnitude of 100 kN, is applied vertically downward at the lower chord nodes 5, 6, 14, 17, 18, 26, and 29.

### 5.1. Vertical Separation Substructuring

**Figure 8:**
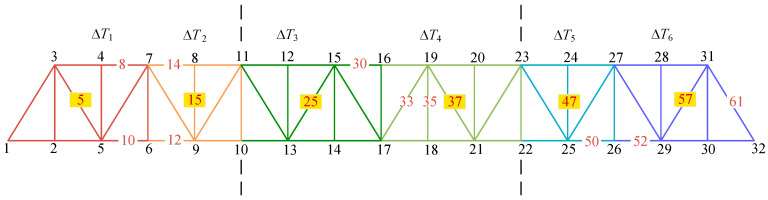
Vertical separation.

For the vertical separation strategy, referred to as Case B, the overall structure was divided into three substructures, with separation locations indicated by black dashed lines in Figure 8. The structure is divided into six longitudinal temperature zones, labeled ΔT_1_ to ΔT_6_ in Figure 8. Two temperature scenarios are considered. In Case B-1, the temperature change increases by 2 °C per zone, from ΔT_1_ = +5 °C to ΔT_6_ = +15 °C relative to the baseline. In Case B-2, the temperature change decreases by 3 °C per zone, from ΔT_1_ = +10 °C to ΔT_6_ = −5 °C. The damaged members for Cases B are summarized in Table 3. Strain measurements are taken at the damaged members. In addition, other strain measurements are located at each temperature zone, with the measurement points marked in yellow, as shown in Figure 8.

**Figure 9:**
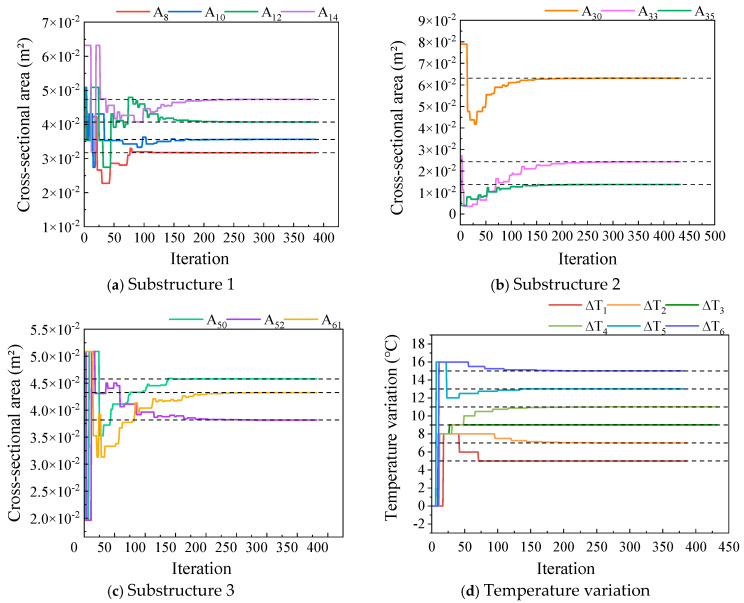
Damage identification results for Case B-1.

**Figure 10:**
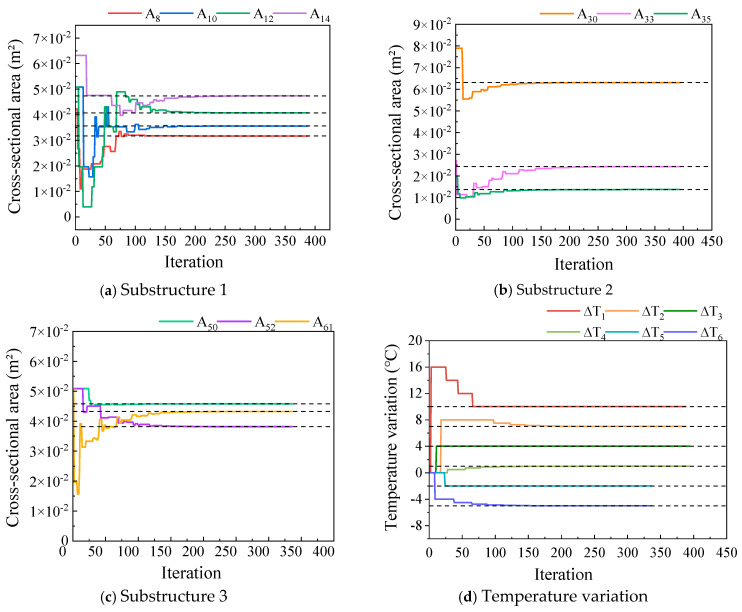
Damage identification results for Case B-2.

**Figure 11:**
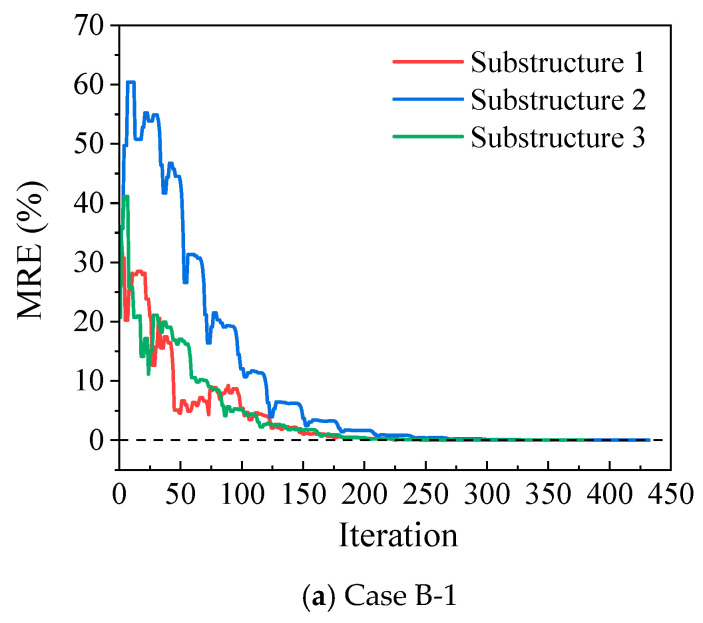

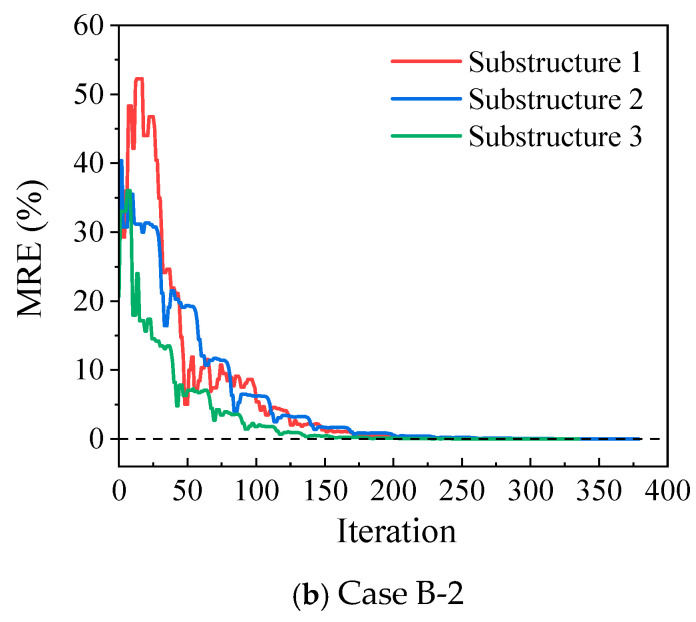
MRE results for Case B.

For each temperature scenario, the cross-sectional areas of the damaged members and the temperature variations were identified simultaneously within each substructure. In Case B-1, the objective functions for Substructures 1, 2, and 3 all converged through the optimization process. The final identification results for each substructure are shown in Figure 9. Figure 9a–c present the iteration process of the damage parameters within Substructures 1, 2, and 3, respectively. Figure 9d shows the iteration process of the temperature parameters merged from the three substructures. Case B-2′s corresponding results are presented in Figure 10. The variation in the MRE with iteration steps during the identification process for both cases is illustrated in Figure 11. These results verify that the proposed method effectively identifies all parameters under combined structural damage and temperature variations.

### 5.2. V-Shaped Separation Substructuring

**Figure 12:**
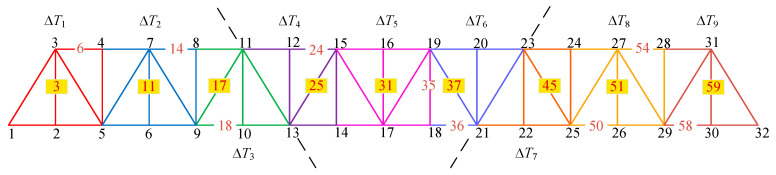
V-Shaped Separation.

In Case C, the whole structure is divided into three substructures via V-shaped separation, as shown in Figure 12. Correspondingly, nine longitudinal temperature zones are defined, labeled ΔT_1_ to ΔT_9_ in Figure 12. Two temperature scenarios are studied. In Case C-1, the temperature change increases by 2 °C per zone from ΔT_1_ = −10 °C to ΔT_9_ = +6 °C. In Case C-2, a symmetric temperature distribution is applied: the temperature change peaks at the central zone ΔT_5_ = +18 °C and decreases by 3 °C per zone towards both ends, reaching +6 °C at ΔT_1_ and ΔT_9_. The damaged members of Case C are presented in Table 4. In this case, strain measurements are also taken at the damaged members. Additionally, the strain measurement points at each temperature zone are marked in yellow in Figure 12.

**Figure 13:**
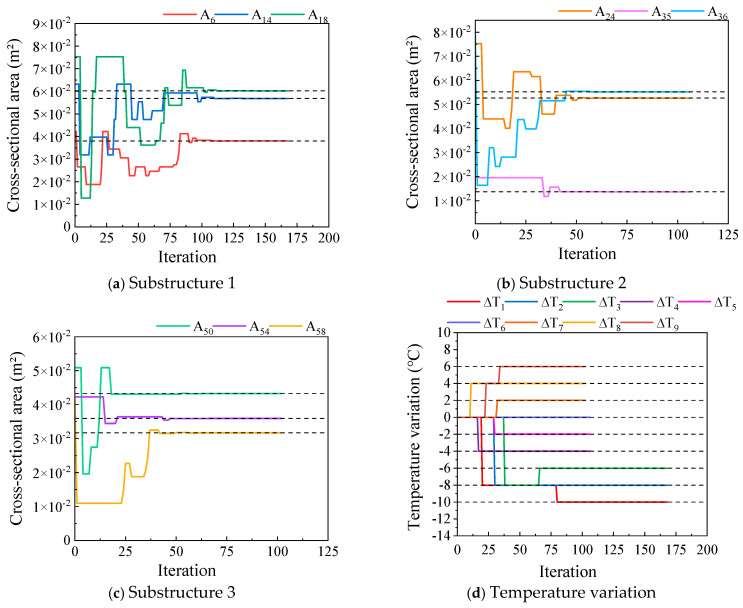
Damage identification results for Case C-1.

**Figure 14:**
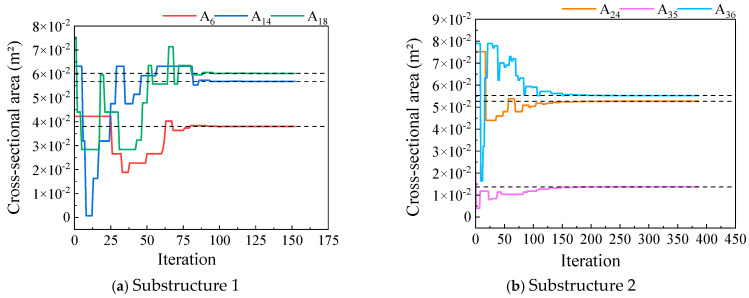

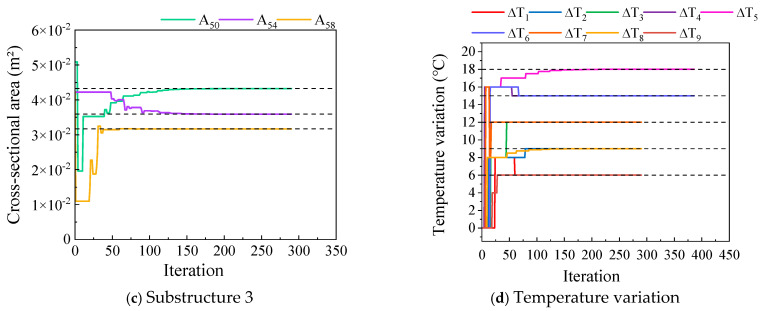
Damage identification results for Case C-2.

**Figure 15:**
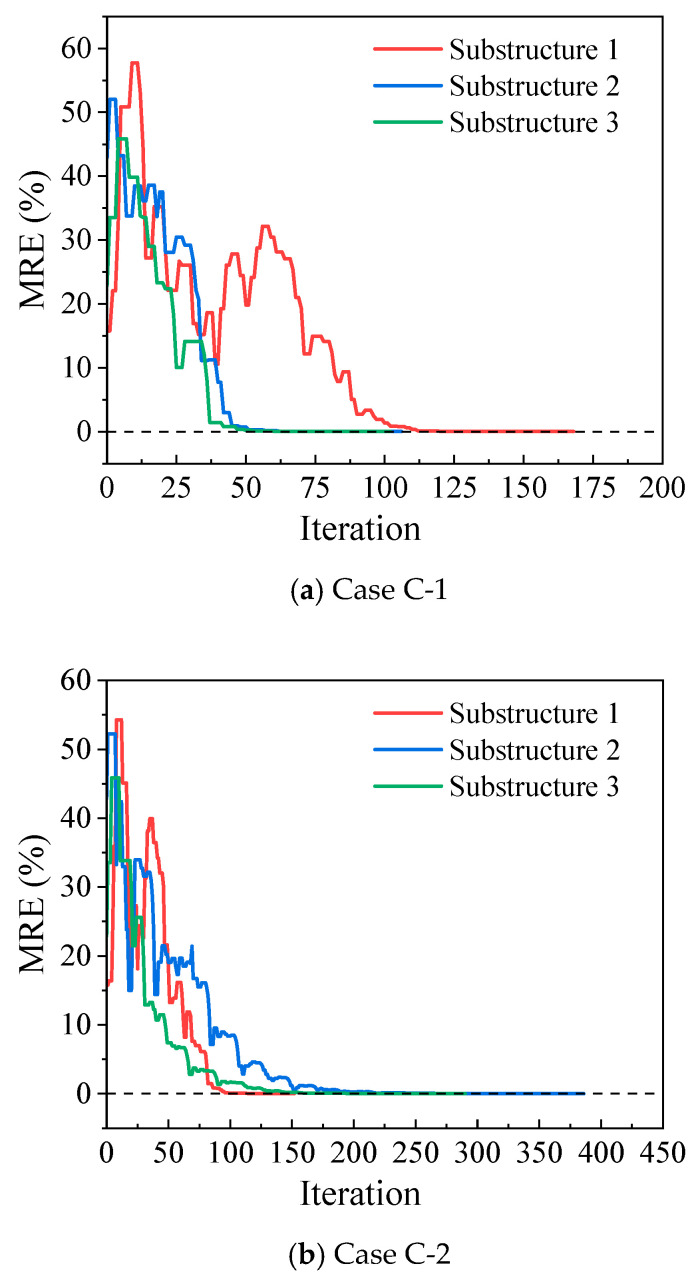
MRE results for Case C.

The optimized identification results for Cases C-1 and C-2 are shown in Figure 13 and Figure 14, respectively. Figure 15 presents the MRE convergence of the identification process. These results further demonstrate the effectiveness of the proposed method.

## 6. Conclusions

This study proposes a damage identification method for truss structures under temperature variations based on the stiffness separation approach. In this method, temperature variations are treated as unknown parameters and are jointly identified with the damage parameters. The method couples the joint damage–temperature identification approach with the stiffness separation method, effectively decoupling the thermal effects from structural damage. Applications to both a Howe-style truss and a truss bridge demonstrate that the proposed method accurately identifies structural damage under the simulated temperature scenarios. The proposed method reduces computational complexity while maintaining high identification accuracy for large, complex structures. These numerical case studies verify the effectiveness of this method under different temperature conditions. In future studies, measurement errors will be introduced to evaluate the robustness of the proposed method, and the extension to other types of structures will be further investigated.

## Figures and Tables

**Table 1:** Damage scenarios for the Howe-style truss.

Damage Case	Damaged Member	Degree of Damage
A-1	4, 12, 18	30%, 25%, 20%
A-2	6, 10, 17	40%, 35%, 25%
A-3	1, 8, 21	35%, 25%, 20%

**Table 2:** Temperature variations in different zones.

Damage Case	Temperature Zone	Temperature Variations (°C)
A-1	$\Delta T_{1}$, $\Delta T_{2}$, $\Delta T_{3}$	8, 12, 15
A-2	$\Delta T_{1}$, ${\Delta T}_{2}$, $\Delta T_{3}$	−3, 7, −2
A-3	$\Delta T_{1}$, $\Delta T_{2}$, ${\Delta T}_{3}$	−5, −10, −15

**Table 3:** Damaged members and cross-sectional areas for Case B.

Damaged Member	Damaged Cross-Sectional Area (×10^−4^ m^2^)
8	316.80
10	356.16
12	407.04
14	473.76
30	631.30
33	243.07
35	137.31
50	457.92
52	381.60
61	432.48

**Table 4:** Damaged members and cross-sectional areas for Case C.

Damaged Member Number	Damaged Cross-Sectional Area (×10^−4^ m^2^)
6	380.16
14	568.51
18	602.11
24	526.85
35	137.31
36	552.38
50	432.48
54	359.04
58	316.80

## Data Availability

Data is contained within the article.
